# Starting a neurosurgical service in a Southern Nigeria rural community. Prospect, challenges, and future—the Irrua experience

**DOI:** 10.1186/s41984-020-00081-y

**Published:** 2020-03-12

**Authors:** E. Morgan, C. Nwatuzor

**Affiliations:** 1 Department of Surgery, Ambrose Alli University, Ekpoma, Nigeria; 2 Neurosurgery Division, Department of Surgery, Irrua Specialist Teaching Hospital, Irrua, Nigeria

**Keywords:** Neurosurgical, Rural, Challenges

## Abstract

Neurosurgical services is an essential component of tertiary level of health care, this field is still evolving in Nigeria with lack of material and manpower. There is a near complete absence of neurosurgeons in the rural communities in Nigeria with over 95% resident in urban area. Starting a neurosurgical services takes a huge sacrifice from the neurosurgeon who is faced with a decision to do something in the presence of a near absence of material and manpower, and in turn circumvent processes with extra burden so as to ensure he/she provides leadership with respect to patients care and assists in the training of allied surgeons and other healthcare staffs to mitigate against morbidity and mortality. Our research is aimed to highlight the total number of patients attended to in the neurosurgical unit, challenges, prospect, and future.

## Introduction

“Neurosurgery is the surgical specialty that treats diseases and disorders of the brain and spinal cord” [1]. “Rural and low-resource societies have diminished capacity to carter for neurosurgical patients due to lack of infrastructure, healthcare investment, and training programs for health care practitioners” [2].

Irrua demographically is a rural community located in Edo Central Local Government area of Edo State whose people’s major occupation is subsistence agriculture. It hosts a federal teaching hospital which serves primarily to the people of Irrua and adjoining rural communities.

### Evolution of neurosurgery

“Modern neurosurgery began in the 19^th^ century when surgeons opened into the meninges. Before then surgeons were doing mainly trepanation. In the 20^th^ century Harvey Cushing introduced new techniques and equipment which was followed with improved outcome. His effort reduced mortality from head and spinal injuries during the world-war II. Advances in cranioplasty emanated from treating wartime casualties.” [3]

“Following the World War II, the need for additional specialized training became obvious. After the war, General surgeons and partially trained neurosurgeons were further trained.” [3] All these are done to further ensure more dedicated manpower to the practice of neurosurgery.

“There are few neurosurgeons in Africa. A recent estimate put the ratio of neurosurgeons in Africa to population at 1 neurosurgeon: 4,000,000 people. This low ratio has made neurosurgical services unavailable to a large section of the population due to several factor ranging from economic, sociocultural and geographical” [4, 5].

“The outlook in Nigeria is even worse with about 27 neurosurgeons serving an estimated population of 170 million (ratio of 1:6.3 million. However, over the last 10 years, about 50 neurosurgeons have been trained and added to this number. In Kenya [6], it is estimated to be about 1 in 3.2 million compared to 1 in 60,000 Americans [7]. The African neurosurgeons are, almost exclusively, in urban centers. As of 2011, a United Nations report estimated that 632 million people, representing 60.4% of the Africa’s population, live in rural areas. Many of these people have poor or no access to neurosurgical care ”[8].

## Main text

### Methodology

Our data emanated from a prospective neurosurgery database over an 18 months period from May 2017-November 2018. Patient data obtained include the following: the biodata, aetiologies of the trauma (road traffic accident, domestic fall, fall from height, gunshot injury to the head, trivial head banging, and others), and other specific neurosurgical pathologies, intervention, and outcome assessment. Pro forma was designed for all categories of anticipated pathologies and consent obtained from patient or relatives. Demographic details with regard to age and sex distribution, presenting pathologies, intervention, and outcome were recorded. Data obtained was analyzed using SPSS version 21 using a simple descriptive statistic tool to present results in table mainly.

## Result

### Demography

We managed a total of 450 patients over the study period, of which 315 were males (70%) and 135 females (30%). Majority of these patients were between 15-45 years (255, 56.7%) (Table 1).

**Table 1:** Demography of cases seen by the neurosurgical unit at the Irrua Specialist Teaching Hospital

**Sex**	**Number**	**Percentage**
Male	315	70
Female	135	30
**Age**	Frequency	Percentage (%)
0-15	68	15.1
> 15-45	255	56.7
> 45-65	95	21.1
> 65	32	7.1

### Statistical summary of cases and treatment

The leading cause of presentation was trauma. Seventy percent of the patients reviewed were male and 30% were female, a finding in agreement with literature pointing to male preponderance [9]. Out of which majority sustained the head and spine injuries. The etiologies of the traumatic brain injury in order of occurrence are road traffic accident from motorcycle accident (41.3%) and motor vehicular (34.8%), domestic fall (14.9%), fall from height (3.3%), gunshot injury to the head (2.3%), and trivial head banging in elderly (3.4%). Majority of these patients sustained mild traumatic brain injury (TBI) and others sustained either moderate or severe TBI which is similar to findings from rural communities in developing countries [10] (Table 2).

**Table 2:** The statistical summary cases seen and other pathologies occasionally seen in Irrua Specialist Teaching Hospital

Pathology	Number	Percentage (%)
**Trauma**		
Head injury		
Mild	100	37.3
Moderate	60	22.3
Severe	58	21.6
Spine injury		
Cervical	22	8.5
Thoracic	10	3.7
Lumbar	8	2.9
Birth trauma	1	0.3
**Congenital malformation**		
Hydrocephalous	10	3.7
Spinal bifida	5	1.8
Myelomeningocele	3	1.1
Encephalocele	1	0.3
**Tumor**		
**Brain**	8	2.9
**Spine**	3	1.1
Spondylosis	5	1.9
Subacute-chronic subdural haematoma	**14**	**5.2**

Of the spine injury cases, majority were cervical spine injuries that were closely followed by lumbar spine injuries. Congenital hydrocephalus constituted a larger number of the congenital malformation. Thirty percent of the patients had operative management. Most were burr hole craniostomy for hematoma and abscess (108, 80%), craniotomy for trauma and tumor excision (10, 7.4%), and others including decompressive craniectomies, elevation of depressed skull fractures, ventriculoperitoneal shunting, and repair of myelomeningoceles and meningocele.

#### Outcome

The median follow-up of patient in our rural setting is 1-12 months. After this period, the outcome is summarized in Table 3.

**Table 3:** Outcome of the intervention

	Outcome		
Procedure	Successful	Death (mortality)	Infected
Burrhole for Haematomas	196 (90%)	21 (10%)	
Burrhole for abscess	16 (80%)	4 (20%)	
Craniotomy for trauma	40 (80%)	10 (20%)	
Craniotomy for tumor excision	5 (100%)	0 (%)	
Decompressive craniectomies	32 (91.4%)	3 (8.6%)	
Ventriculoperitoneal shunt	15 (75%)	2 (13%)	3 (15%)
Repair of meningocele	2 (100%)	0	0

Table 4 summarizes the outcome of TBI using GOS with majority good outcome and 20 out the 218 patients with TBI dying during the course of treatment. None of the patient had a GOS of 2.

**Table 4:** Glasgow outcome scale (GOS) within 6 months management of TBI

GOS	Number	Percentage (%)
Good	112	51.3
Moderate disability	50	22.9
Severe disability	36	16.5
Death	20	9.2
Total	218	100

## Discussion

Irrua Specialist Teaching Hospital is geographically located in a rural community with a population of about 200,000. Starting a neurosurgical service in a rural setting portend a great challenge and it is tasking, our setup has also been likened to other similar settings as seen in the research by Rabiu et al. [11] at Oshogbo, Nigeria, where the following were also encountered.

### Challenges

The challenges of starting a neurosurgical center in the rural area are quite enormous, though there are paucity of literature [2, 8, 11] on these challenges. These challenges include, ineffective coordination between all health care staffs with significant time wasted in attending to patients, manpower, critical care, materials for intervention, limited theater space, and post-operative care and which can result in significant mortality due to a variety of factors discussed below:

### Manpower

There are few neurosurgeon practicing in the rural area in Nigeria, and this is the usual trend worldwide as earlier pointed out in literatures [2, 3, 8]. As earlier studies have identified, resulting in the referral of complex cases to urban area which has a poor transportation network and communication facilities with the rural area with significant chances of morbidity/mortality. In our rural setup, there is only one neurosurgeon attending to a population of over 200 thousand people with no trained support staffs such as neuro nurses, neuro physiotherapist, and neuro physiology. This creates a cumbersome work environment for the neurosurgeon who must interface among this specialties and in turn train local manpower to meet up with need. Studies from several low income countries have also pointed out this factor as responsible for great death of morbidity and mortality [12], even leading to the instance of training general surgeons and orthopedic surgeons to be able to handle some neurosurgical cases. Our experience of the need for manpower stemmed from a situation where long waiting list with paucity of support staffs and other relevant specialist unavailability due to their limited number, and attending to other cases.

### Critical care

The lack of resources to offer a succinct critical care is the first among the numerous challenges in our setting. These include human resources, equipment, and intensive care unit. Our intensive care unit for instance has only two ventilators which sometimes are non-functional. We do not have a mobile X-ray making unstable patients to be wheeled some meters away from the emergency point to get radiological investigations. Oxygen pipeline not guaranteed. It can be interrupted anytime. Mobile oxygen is not readily available. These problems have also been identified in other low income countries [12], and thus correlate with morbidity and mortality. These are also the absence of post-operative neuromonitoring and haemodynamic device, and thus has great impact on patients care. These issues were also noted some literature [8, 11]. This sometimes endangers the patient, worsens neurological state of the patient, and overall outcome.

### Materials for surgical intervention

The materials for neurosurgical procedures are not readily available in the rural setting with ours not been an exception. Most are gotten from the city. This eventually causes delay in intervention predisposing to a frequency of mortality as recorded in Tables 3 and 4. The cost of the items is further increased as well as putting strain on these local inhabitant who live in less than 2 dollars per day.

### Operating theater

Only three suits are available for both elective and emergency cases. With busy elective list, emergency cases are occasionally delayed and (in few cases are done in suite where other procedure which are however not for clean cases as shown in Table 3 where we had three cases of shunt tract infection out of which two died), thus resulting in morbidity and mortality. Another confounding factor is the non-availability of operating day for neurosurgery in our center causing significant delay in the review and intervention of neurosurgical patients. This leaves the unit with very few elective cases. Appropriate surgical instruments are not provided by the hospital in the government extension. This leaves the surgeon with no choice but to acquire his own equipment to ease his services which thus affects patient care.

### Post-operative challenges

The role of the intensive care unit and functional mechanical ventilator in the post-operative management of neurosurgical patients cannot be over emphasize, few ventilator support thus resulting in the hospital prolong waiting list, makes optimal emergency care unattainable, and increases the chances of morbidity and mortality In the same vein, absent neuromonitoring devices and lack of neurosurgical trained support staffs affect the outcome of management of patients in the rural community.

## Outcome

Table 3 shows the pattern of outcome of various interventions. Twenty-one out of 217 died following burr hole craniostomy for hematoma, and all presented with poor neurological status. Ten out of 50 died following craniotomy for trauma and 3 have shunt tract infection out of which 2 died. This pattern out outcome mirrored finding from other rural setting where there are delay in attending to patients coupled with paucity of materials and manpower [13]. Majority of our mortality emanated from absence of neuromonitoring devices and adequately manned and equipped intensive care unit. For patients with TBI, the majority 112 (51.3%) out of 218 cases had good outcome and could return to pre-morbid activities of daily living. Twenty (9.2%) out of 218 died during the course of the treatment as shown in Table 3.

### Prospect and future way forward

The future of neurosurgery in the rural community is bright if all these highlighted problems are addressed through a collaborative effort of government, donor agencies, and public and private individuals. It is imperative that the first approach in improving the practice of neurosurgery in rural settings is conceptualization and implantation of the setup. Setting up neurosurgical services includes training of neurosurgeons and allied health staff as was done in the Peruvian setup [14]. Subsequently is sequential provision of materials for neurosurgical services with regard to provision of good transportation services, means of communication, readily provision of equipment to aid investigation (imaging modalities such as computerized tomography scan and magnetic resonance imaging) and equipment intervention for both basic and complex neurosurgical cases as well as a proper intensive care unit with neuromonitoring facilities. If all these steps are followed and put in place, neurosurgical service will be at the doorstep of the rural dweller and thus reduced morbidity and mortality currently encountered on account of paucity of manpower and materials for effective service delivery. Training and re-training of other allied health workers like nurses and physiotherapist who are directly or indirectly involved in the management of a neurosurgical patient.

## Conclusion

Neurosurgical services are of paramount importance in rural and urban areas. Mortality tends to be higher in the rural setting in view of deficit of manpower and materials. These challenges are colossal calling for a collaborative effort by all and sundry, starting conceptualization, staffing, and patients’ care right from the point of arrival at the emergency room, to the theater and up to point of discharge and follow-up.

## Data Availability

Applicable

## Abbreviations

CT: Computed tomography
GOS: Glasgow outcome scale
ICU: Intensive care unit
TBI: Traumatic brain injury
